# Corrigendum to: Relationship Between Odor Intensity Estimates and COVID-19 Prevalence Prediction in a Swedish Population

**DOI:** 10.1093/chemse/bjaa042

**Published:** 2020-07-07

**Authors:** Behzad Iravani, Artin Arshamian, Aharon Ravia, Eva Mishor, Kobi Snitz, Sagit Shushan, Yehudah Roth, Ofer Perl, Danielle Honigstein, Reut Weissgross, Shiri Karagach, Gernot Ernst, Masako Okamoto, Zachary Mainen, Erminio Monteleone, Caterina Dinnella, Sara Spinelli, Franklin Mariño-Sánchez, Camille Ferdenzi, Monique Smeets, Kazushige Touhara, Moustafa Bensafi, Thomas Hummel, Noam Sobel, Johan N Lundström

Chem. Senses (2020). doi:10.1093/chemse/bjaa034.

This is a correction notice for article bjz034 (DOI: https://doi.org/10.1093/chemse/bjaa034), published on 22 May 2020. Due to an error in the script used to create subsections of Figure 1, there was both a shift in the intensity data and an erroneous calculation of error bars in all panels. Figure 1 and the accompanying figure legend have been revised to show the correct levels and error bars. This script error only affected visualization of the data in Figure 1 and did not impact the reported data or conclusions.

**Figure 1.**
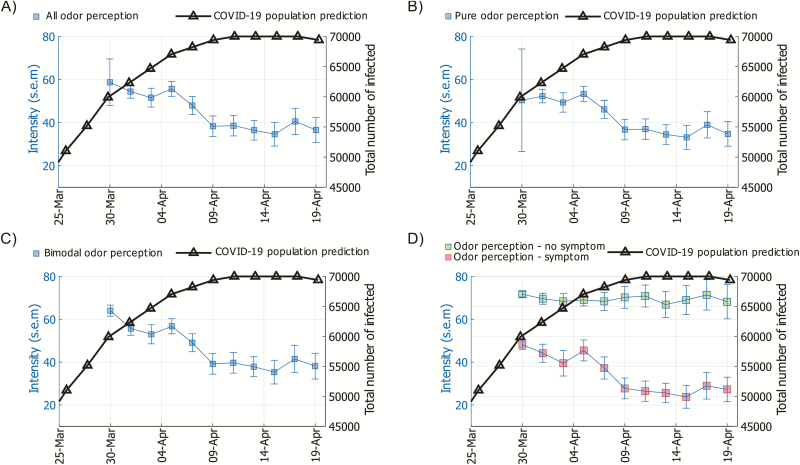
Odor intensity perception relate to COVID-19 prevalence. **(A)** Mean intensity ratings of the 5 odor categories (blue line and axis) in relation to population prediction (black line and axis) of COVID-19 prevalence in the Stockholm region. **(B)** Mean intensity ratings of unimodal odors (odor categories 1 and 2; blue line and axis) in relation to population prediction of COVID-19 prevalence in the Stockholm region. **(C)** Mean intensity ratings of bimodal odors (odor categories 3–5; blue line and axis) in relation to population prediction of COVID-19 prevalence in the Stockholm region. (D) Mean intensity ratings of odors (categories 1–5), separated into individuals without (green squares, blue axis) and with (purple squares, blue axis) reported COVID-19 symptoms, in relation to population prediction (black line and axis) of COVID-19 prevalence in the Stockholm region. Error bars in all panels indicate standard error of the mean (SEM). Error bars for first day of testing are large due to few participants that day.

