# Supplementary material for: Relationship Between Odor Intensity Estimates and COVID-19 Prevalence Prediction in a Swedish Population
Source: Chem Senses. 2020 May 22:bjaa034. doi: 10.1093/chemse/bjaa034 (PMC7314115; doi:10.1093/chemse/bjaa034)
Supplement: bjaa034_suppl_Supplementary_figures [file bjaa034_suppl_supplementary_figures.pdf]

Supplementary table & figures

Relationship between odor intensity estimates and COVID-19 prevalence prediction in a Swedish population

----

	30-Mar	01-Apr	03-Apr	05-Apr	07-Apr	09-Apr	11-Apr	13-Apr	15-Apr	17-Apr	19-Apr
Fever	24	99	39	74	40	45	51	55	29	24	24
Cough	67	108	49	99	74	61	71	63	41	29	34
ShortnessOfBreathOrDifficultyBreathing	28	100	39	81	49	54	45	53	30	28	19
Tiredness	80	117	50	99	73	61	74	69	41	40	32
Aches	41	107	41	84	53	57	61	61	33	28	31
RunnyNose	62	110	48	87	68	68	65	64	38	33	28
SoreThroat	49	122	43	94	57	51	57	52	36	27	28

Counts (color scale: 0, 20, 40, 60, 80, 100, 120)

Supplementary Table S1. Count of symptoms indicated by individuals who reported having symptoms of COVID-19, separated by mean testing date. Colors indicate number of values in each cell.

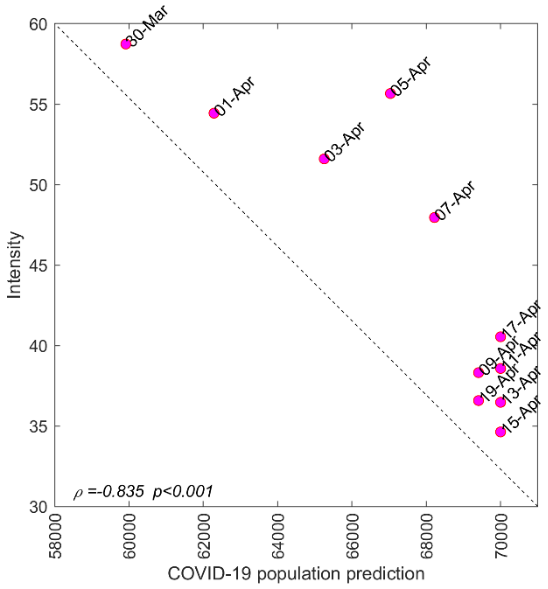

Supplementary Figure S1. Relationship between Intensity rating over time and the COVID-19 population prevalence model, plotted over dates.

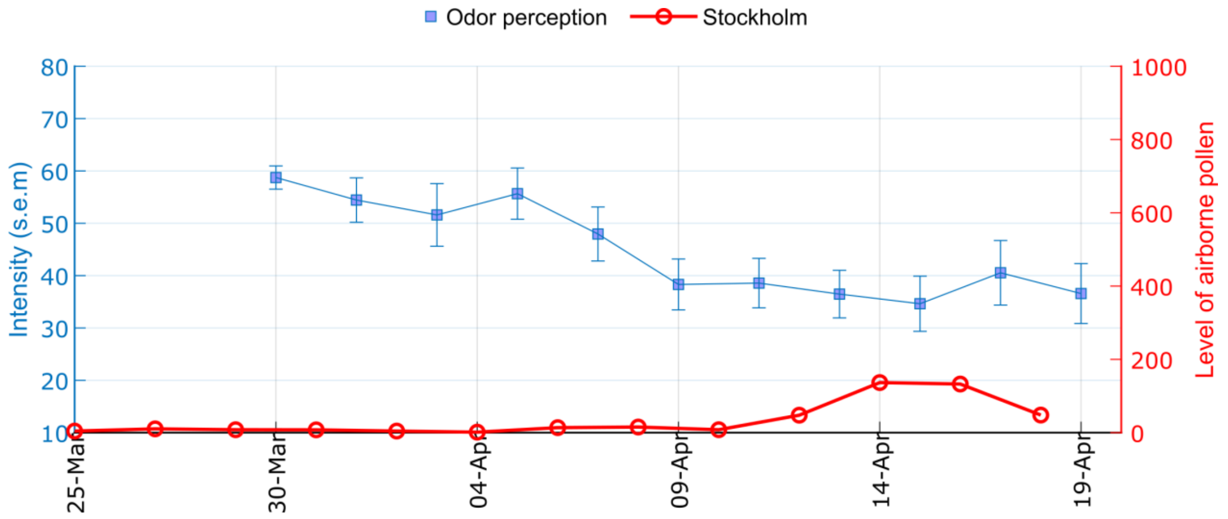

Supplementary Figure S2. Relationship between Intensity rating over time and summated levels of allergenic airborne pollen in the Stockholm area. Blue line and axis indicate mean intensity rating over time and red line and axis summated levels of detected airborne pollen.

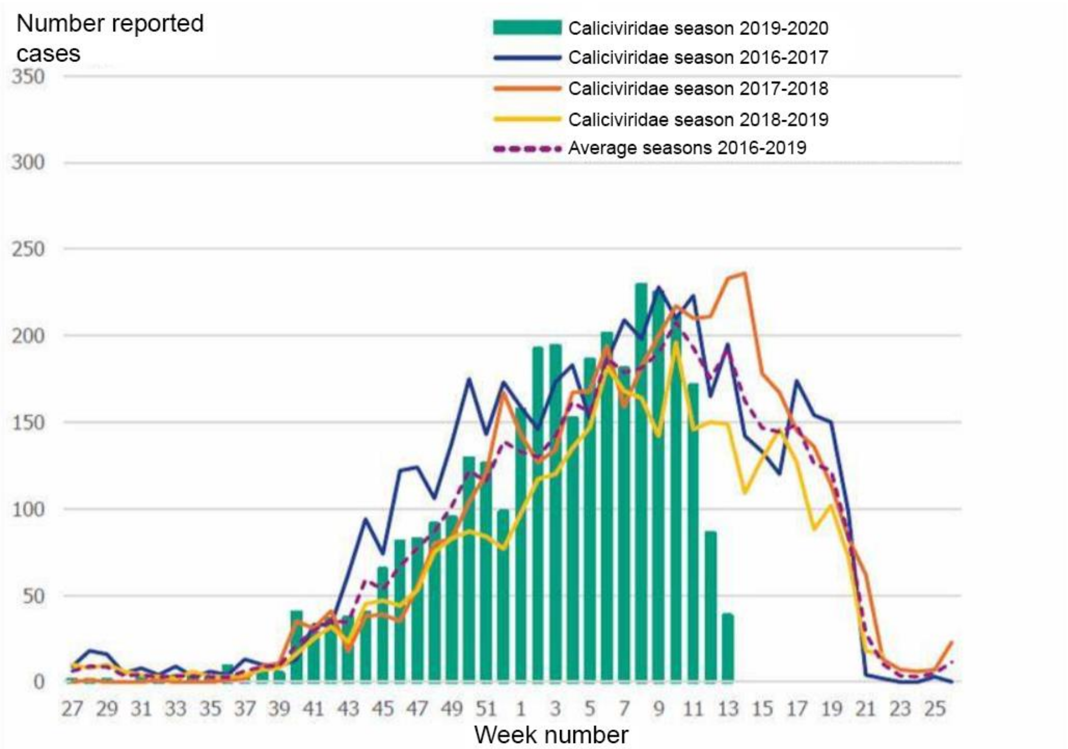

Supplementary Figure S3. Number of reported laboratory cases for confirmed caliciviridae virus (gastroenteritis), in Sweden known as the Winter vomiting disease, and a common transmittable seasonal disease in Sweden. Note that week 12 (the first major deviation from norm) is 9-15/3. Data collected for this manuscript started in week 18 (>30/4, 2020).
